# Return to work in patients with lumbar disc herniation undergoing fusion

**DOI:** 10.1186/s13018-021-02682-1

**Published:** 2021-08-27

**Authors:** Lauren A. Protzer, Steven D. Glassman, Praveen V. Mummaneni, Mohamad Bydon, Erica F. Bisson, Mladen Djurasovic, Leah Y. Carreon

**Affiliations:** 1 Department of Orthopaedic Surgery, University of Louisville School of Medicine, 550 S. Jackson Street, 1st Floor ACB, Louisville, KY 40202 USA; 2 Norton Leatherman Spine Center, 210 East Gray Street, Suite #900, Louisville, KY 40202 USA; 3 Department of Neurological Surgery, University of California, San Francisco, 505 Parnassus Ave. Rm. M779, San Francisco, CA 94143-0112 USA; 4 Department of Neurologic Surgery, Mayo Clinic, Rochester, MN USA; 5 Department of Neurosurgery, University of Utah Health, 175 N. Medical Drive East, 5th Floor, Salt Lake City, UT 84132 USA

**Keywords:** Lumbar disc herniation, Lumbar fusion, Discectomy, Return to work, QOD, Quality Outcomes Database

## Abstract

**Background:**

Lumbar disc herniation (LDH) is a common problem. When surgical treatment is required, the intervention is typically decompression without fusion. Successful return-to-work (RTW) is a standard expectation with these limited procedures. Occasionally, the size or location of the disc herniation suggests the need for fusion, but the inability to RTW is a significant concern in these cases. The purpose of this study is to determine if the addition of lumbar fusion, as compared to decompression alone, will substantially diminish RTW in patients with lumbar disc herniation.

**Methods:**

This is a longitudinal cohort study using prospectively collected data from the Quality and Outcomes Database (QOD). Patients with LDH, eligible to RTW (not retired, a student, or on disability) with complete 12-month follow-up data, were identified. Standard demographic and surgical variables, patient-reported outcomes (PROs), and RTW status at 3 and 12 months were collected.

**Results:**

Of the 5062 patients identified, 4560 (90%) had decompression alone and 502 (10%) had a concurrent fusion. Age and gender were similar in the two groups. The fusion group had worse back pain (NRS 6.52 vs. 5.96) and less leg pain (6.31 vs. 7.01) at baseline compared to the no fusion group. Statistically significant improvement in all PROs was seen in both groups. RTW at 3 months post-op was seen in 85% of decompression cases and 66% of cases with supplemental fusion. At 12 months post-op, RTW increased to 93% and 82%, respectively.

**Conclusion:**

The need for fusion in LDH cases is unusual, seen in only 10% of cases in this series. The addition of fusion decreased the RTW rate from 85 to 66% at 3 months and from 93 to 82% at 12 months post-op. While the difference is significant, the ultimate deterioration in RTW may be less than anticipated. A reasonable RTW rate can still be expected in the rare patient who requires fusion as part of their treatment for LDH.

## Introduction

Lumbar disc herniation (LDH) is a common pathology which is responsible for 20% of low back pain leading to disability and work absence [1]. The incidence of a herniated disc is 5 to 20 cases per 1000 adults annually. It is most common in people in their third to the fifth decade of life, with a male to female ratio of 2:1 [2]. Eighty-five percent of patients with an acute LDH improve spontaneously with conservative management [3]. NSAIDs and physical therapy are the first-line treatment modalities, followed by epidural injections [4].

When surgical treatment is required for a LDH, this usually occurs after at least 6 weeks of failed conservative treatment. The intervention is typically partial discectomy without fusion to alleviate radicular symptoms. Occasionally, the size or location of the disc herniation suggests a need for fusion. A far lateral disc herniation may require a significant resection of the facet joint, rendering that level unstable. There may be instances in which the decompression alone results in a significant loss of disc height. As the intervertebral disc is considered to be a biomechanical stabilizer of the spinal unit, the surgeon therefore may decide to proceed with fusion. Segmental instability after a discectomy is thought to be one of the risk factors for residual low back pain and recurrence of disc herniation [5, 6]. There may also be instances in which the patient might not be deemed to require fusion by most surgeons, but fusion is undertaken due to individual surgeon preference. Prior studies have examined the influence of financial incentives in the surgical decision-making process [7].

Successful return-to-work (RTW) is a standard expectation with decompression alone, whereas inability to RTW is a significant concern in those cases requiring decompression with fusion. To our knowledge, no study has compared the RTW of patients who underwent a decompression without fusion compared to a decompression with spinal fusion in LDH patients. The purpose of this study is to determine if the addition of lumbar fusion, as compared to decompression alone, will substantially diminish RTW 1 year after surgery in patients with LDH.

## Materials and methods

The Quality Outcomes Database (QOD) registry was queried in December 2018 for patients with lumbar disc herniation (LDH) who underwent operative treatment with either a decompression alone or a decompression with fusion. QOD is a prospective registry, established in 2012, that was designed to evaluate risk-adjusted expected morbidity and 12-month outcomes with the goal of providing insights into and ameliorating the efficiency and quality of care for the most commonly performed spinal surgical procedures in the USA [8, 9].

Patients who were included in the study were eligible to return to work (RTW). Those who were identified as retired, a student, or on disability were excluded. Other inclusion criteria were age greater or equal to 18, at least 1-year follow-up from surgery, and the procedure performed as a primary operation and not for a recurrent disc herniation.

Standard demographics included age, sex, BMI, and surgical variables including estimated blood loss, and total operating room time was also evaluated. Patient-reported outcomes (PROs) at baseline, 3 and 12 months, were collected including back and leg pain numeric rating score (NRS) [10], Oswestry Disability Index (ODI) [11, 12], and RTW and if there were any associated worker’s compensation claims.

All statistical analysis was performed using IBM SPSS Statistics for Windows, Version 27.0 (Armonk, New York). Baseline characteristics of the decompression alone and the fusion groups were compared using unpaired independent *t* tests for continuous data and Fisher’s exact test for categorical variables. Repeated measures ANOVA with the baseline PRO score as a co-variate was used to compare PRO changes over time between the two groups. Fisher’s exact test for categorical variables was used to compare return to work rates between the two groups at 3 and 12 months after surgery. Binary logistic regression was performed to identify variables associated with return to work at 12 months. Statistical significance was set at *p*<0.01.

## Results

Of the 5062 patients meeting inclusion criteria, 4560 (90%) had decompression alone and 502 (10%) had a concurrent fusion (Table 1). Age, gender, and BMI were similar in the two groups. The proportion of patients receiving Workers Compensation and the type of work prior to surgery was similar between the two groups. The fusion group had worse back pain (NRS 6.52 vs 5.96, *p*<0.001) but less leg pain (6.31 vs 7.01, *p*<0.001) at baseline compared to the no fusion group. EQ-5D and ODI scores were similar between the two groups.

**Table 1:** Summary of baseline demographic and clinical outcomes data

	No fusion	Fusion	
*N*	4560	502	
Age, years, mean (SD)	47.43 (12.10)	48.61 (11.07)	0.038
Males, *N* (%)	2798 (61%)	294 (59%)	0.177
BMI, kg/m [2], mean (SD)	29.87 (6.23)	30.37 (5.96)	0.010
Worker’s compensation, *N* (%)	375 (8%)	39 (1%)	0.689
Type of work prior to surgery, *N* (%)			0.063
Sedentary	1584 (35%)	144 (29%)	
Light	1120 (25%)	128 (25%)	
Medium	1051 (23%)	143 (28%)	
Heavy	1584 (35%)	144 (29%)	
Baseline scores, mean (SD)			
Back pain	5.96 (2.89)	6.52 (2.41)	<0.001
Leg pain	7.01 (2.53)	6.31 (2.77)	<0.001
EuroQOL-5D	0.54 (0.22)	0.56 (0.22)	0.158
Oswestry Disability Index	47.39 (17.51)	47.67 (14.95)	0.688

The OR time was significantly greater in the fusion (172.53 min) vs the no fusion (75.54 min, *p*<0.001) group. The fusion group also had significantly more blood loss (249.44 mL) than the no fusion (49.82mL, *p*<0.001) group. Repeated measures ANOVA showed that the fusion group also had worse PROs through 3 and 12 months compared to the no fusion group even when adjusting for the differences in the baseline pain scores. The ODI was statistically similar at baseline in both groups (fusion 46.67 vs no fusion 47.39, *p*=0.688), and although both groups improved substantially, the fusion group demonstrated less improvement vs. the no fusion group at 12 months (23.28 vs 17.21, *p*=0.004).

At 3 months, 85% of the decompression-only patients had returned to work compared to 66% of the fusion group (*p* <0.001). At 12 months, this difference was still significant, decompression only group had 93% RTW and fusion only 82% RTW (*p* <0.001). Binary logistic regression analysis showed that type of employment prior to surgery, the performance of the fusion, and 12-month EQ-5D were associated with return to work at 12 months after surgery (Table 2). Age, gender, BMI, workers’ compensation, and baseline PRO scores were not associated with return to work at 12 months.

**Table 2:** Summary of surgical and follow-up data

Operative time, minutes, mean (SD)	75.54 (37.21)	172.53 (77.09)	<0.001
Estimated blood loss, mL, mean (SD)	49.82 (85.03)	249.44 (255.85)	<0.001
Back pain, mean (SD)			<0.001
3 months	2.40 (2.51)	3.44 (2.53)	
12 months	2.57 (2.68)	3.33 (2.79)	
Leg pain, mean (SD)			<0.001
3 months	2.02 (2.63)	2.48 (2.86)	
12 months	2.12 (2.79)	2.78 (3.00)	
EuroQOL-5D, Mean (SD)			<0.001
3 months	0.82 (0.18)	0.76 (0.20)	
12 months	0.83 (0.20)	0.77 (0.21)	
Oswestry Disability Index, Mean (SD)			0.004
3 months	18.73 (17.96)	26.54 (18.59)	
12 months	17.21 (18.65)	23.28 (19.52)	
Return to work, *N* (%)			
3 months	3864 (85%)	330 (66%)	<0.001
12 months	4231 (93%)	410 (82%)	<0.001

## Discussion

To our knowledge, this is the first study assessing RTW following decompression versus decompression with fusion for patients with primary LDH refractory to conservative treatment. Decompression alone is obviously the standard treatment in these cases, and only 10% of our study population underwent fusion. This nonetheless represents a substantial population for which the expected RTW rate is not well defined.

Several prior studies have examined RTW in the LDH population, but none have compared decompression only vs. fusion cases. Atarod et al. [13] reviewed 142 patients who underwent lumbar discectomy to determine the rate and contributing factors of return to work in the postoperative phase. They had 113 (79.5%) of patients return to work in 3 months. Predictive factors for RTW included male gender, higher literacy, non-manual job, non-smoker, formal work agreement, normal BMI, lower working hours, and higher income. Than et al. [14] followed 127 patients 3 months postoperatively from lumbar discectomy, and after controlling for patients who were working preoperatively (105 patients), only younger age was a statistically significant predictor of postoperative return to work (44.1 years vs 51.1 years, *p* = 0.049). In the present study, less physically demanding job, performance of a fusion, and 12-month EQ-5D scores were associated with return to work at 12 months. Although 12-month ODI scores were significantly associated, the odds ratio is close to 1.0 which denotes clinical irrelevance. In the present study, 85% of the decompression only patients had returned to work at 3 months post-op as compared to 66% of the fusion group (*p* <0.001). By 12 months post-op, this difference was diminished, but still significant with 93% RTW in the decompression group and 82% RTW in the fusion group (*p* <0.001).

Historically, back pain preoperatively along with radicular symptoms was an indication to perform concomitant fusion with decompression [15]. This is no longer the case, and decompression alone is now the standard treatment for LDH. However, fusion is still a useful adjuvant procedure in the properly screened patient. Vaughan et al. [16] evaluated the results of 85 patients, 52 with discectomy and 33 with discectomy and fusion and determined that the fusion group had significant better results compared to the non-fusion group (85% vs 39%). The only statistical significance in their preoperative values included duration of back pain (fusion 44.8 vs no fusion 23.3 months, *p* < .001), history of chronic back pain > 6 months (fusion 94% vs no fusion 52%, *p* <0.001), percentage of patients positive straight leg raise (fusion 88% vs no fusion 100%, *p* <0.02), and percentage narrow of L4–L5 disc space (fusion 76% vs no fusion 32%, *p* <0.001). The most common cause of unsatisfactory results in the fusion group was pseudarthrosis [2] while progressive degenerative disc disease [18] and recurrent disc prolapse [8] were the most common causes of unsatisfactory results in the discectomy group. Takeshima et al. [17] looked at 95 patients with lumbar disc herniation requiring surgery, 45 patients had discectomy, while 51 underwent discectomy and fusion. Clinical outcome was excellent or good in 73% of the non-fusion group and 82% of the fusion group (*p* =0.31), and patients with fusion had a greater reduction in back pain. This study suggests that if an appropriate patient is selected for fusion for LDH, they could have more positive outcomes.

Satoh et al. [5] looked at 498 patients with a minimum 5-year follow-up who underwent primary surgery at a single level for LDH. They set criteria for fusion as massive herniation and segmental instability. A massive herniation was defined as a complete block on myelogram, and segmental instability was defined as an anterior slip of > 3 mm or local kyphosis of > 5° on a lateral radiograph of maximal flexion. They had four groups: group one underwent fusion and met criteria for fusion; group two had an indication for discectomy only, but underwent fusion; group three had an indication for fusion but underwent discectomy only; and group four had an indication for a discectomy and underwent discectomy. There was no statistical significant difference in any group regarding outcomes of leg pain or low back pain.

The present study is not without limitations. First, this study is a retrospective analysis of prospectively collected registry data and holds the associated limitations. Secondly, our metric of employment status and RTW may lack granularity. Patients only able to return to part-time employment or modified duty remain a significant economic burden to employers, among others. Occasionally, non-physical factors such as motivation, job satisfaction, and financial advantages would have more effect on the decision to RTW than overall back pain and recovery [18]. There are also post-operative factors that are not accounted for in this data. Carragee et al. [19] lifted all post-operative activity restrictions on patients after microdiscectomy, which may also allow those patients to RTW faster. Routine practice with fusion typically limits bending, lifting, and twisting in patients who recently underwent fusion for up to 3 months.

Patients who underwent fusion in our study also had more back pain both pre- and post-operatively and less leg pain at baseline but worse leg pain at 12 months post-op compared to the non-fusion group. The back pain could be related to their original LDH or to a myriad of pre/post-patient factors that are not accounted for in this analysis. Wang et al. [20] evaluated 16/221 (7.2%) patients who experienced persistent low back pain following spinal fusion for LDH with a numeric rating scale (NRS) >50 at all post-operative follow-up time points. They determined that preoperative back pain (NRS >3.5), surgery segment at L5-S1, and large fatty infiltrate rate of the paraspinal muscles (>15%) were significant and independent factors associated with persistent back pain following decompression and fusion. These specific factors were not accounted for in our study and another limitation when comparing the fusion to the no fusion group.

In summary, although the differences in return to work in our decompression vs. fusion cohorts were statistically significant, by 12 months, the difference was relatively small (93% vs 82%). Return to work was slower in the fusion group, but ultimate return to work was better than most prior reports.

The need for fusion in LDH is unusual, only present in 10% of cases in this series. The addition of fusion does decrease RTW from 85 to 66% at 3 months and from 93 to 82% at 12 months postoperatively. While the difference is statistically significant, the value is only modestly impaired. Reasonable RTW can be expected in the rare patient who requires fusion for LDH, and providers should still use their clinical judgment to provide the best treatment option for each patient with the understanding that RTW rates should be acceptable.

## Data Availability

The data that support the findings of this study are available from the NeuroPoint Alliance but restrictions apply to the availability of these data, which were used under license for the current study, and so are not publicly available. Data are however available from the authors upon reasonable request and with permission of the NeurPoint Alliance.

## Abbreviations

RTW: Return to work
LDH: Lumbar disc herniation
QOD: Quality Outcomes Database
PROs: Patient-reported outcomes
NRS: Numeric Rating Scale
NSAIDS: Non-Steroidal Anti-Inflammatory Drugs
BMI: Body mass index
ODI: Oswestry Disablity Index
EQ-5D: EuroQOL 5 Dimenstions
ANOVA: Analysis of variance
OR: Operating room
